# Diagnostic Value of Urine Tissue Inhibitor of Metalloproteinase-2 and Insulin-Like Growth Factor-Binding Protein 7 for Acute Kidney Injury: A Meta-Analysis

**DOI:** 10.1371/journal.pone.0170214

**Published:** 2017-01-20

**Authors:** Yuanyuan Su, Zhiyan Gong, Yan Wu, Yuan Tian, Xiaohui Liao

**Affiliations:** 1 Department of Nephrology, The Second Affiliated Hospital, Chongqing Medical University, Chongqing, China; 2 Department of Ultrasonography, The First Affiliated Hospital, Chongqing Medical University, Chongqing, China; Centre Hospitalier Universitaire Vaudois, FRANCE

## Abstract

**Background:**

Tissue inhibitor of metalloproteinase-2 (TIMP-2) and insulin-like growth factor-binding protein-7 (IGFBP7) are both involved in renal tubular epithelial cell cycle arrest in acute kidney injury (AKI). Several recent studies showed that urine TIMP-2 times IGFBP7 ([TIMP-2]*[IGFBP7]) is a promising biomarker to predict AKI.

**Methods:**

The aim of this meta-analysis was to assess the diagnostic value of urine [TIMP-2]*[IGFBP7] for early diagnosis of AKI. Relevant studies were retrieved from the PubMed, EMBASE, and Cochrane Library databases. The sensitivity and specificity were determined, and summary receiver operating characteristic (SROC) curves were constructed.

**Results:**

Ten full-text prospective studies were included in this meta-analysis. The estimated sensitivity of urine [TIMP-2]*[IGFBP7] for the early diagnosis of AKI was 0.84 (95% CI = 0.80–0.88) and the specificity was 0.57 (95%CI = 0.55–0.60). The SROC analysis showed an area under the curve of 0.8813.

**Limitation:**

The limited number of included studies, small sample size, unpublished negative results and language limitation might have affected the evaluation.

**Conclusion:**

Urine [TIMP-2]*[IGFBP7] is a promising candidate for early detection of AKI, especially in ruling-out AKI. However, the potential of this biomarker should be validated in larger studies with a broader spectrum of clinical settings.

## Introduction

Acute kidney injury (AKI) is a common and serious clinic syndrome that presents as a rapid decrease in kidney function over a short period of time and is associated with a high risk of morbidity and mortality. Various factors that are known to induce AKI include cardiac surgery, infection, and drug toxicity, among others. The morbidity rate of AKI is about 5% among inpatients and 30%-50% among those in the intensive care unit (ICU). Among patients with AKI, about 6% will require dialysis, which is associated with a mortality rate of 60% [1–3]. The prognosis of AKI is dependent on early diagnosis and treatment. Therefore, early diagnosis is a key to successful AKI treatment [4].

Serum creatinine and urine output are major clinical indicators of AKI. However, several other factors, such as muscle volume and diuretic use, also impact these indicators. Serum creatinine levels increase with a decrease in glomerular filtration rate, but in the early stage of AKI, which is mainly caused by tubular injury and necrosis, serum creatinine levels do not reflect renal damage in time for an early diagnosis because an increase in serum creatinine occurs from several hours to as late as 2–3 days after the onset of AKI [5, 6]. Thus, the value of serum creatinine for early diagnosis of AKI is very limited. Although urine output is a good indicator of AKI, renal damage without oliguria render this criterion less reliable [7]. Hence, the identification of a more suitable biomarker has become a central issue to improve early diagnosis and management of AKI.

Neutrophil gelatinase-associated lipocalin (NGAL) and kidney injury molecule-1 (KIM-1) are released by injured tubular epithelium cells, and interleukin-18 (IL-18) is an inflammatory mediator released during AKI. These molecules, which take part in the early pathophysiological changes in AKI, have been recently evaluated as potential new biomarkers. Tissue inhibitor of metalloproteinase-2 (TIMP-2) and insulin-like growth factor binding protein-7 (IGFBP7) both act to block the G1 stage of the renal tubular epithelial cell cycle during AKI [8–11]. A study by Kashani et al. [11] evaluated the diagnostic value of more than 300 biomarkers for early detection of AKI. In that study, the area under the receiver-operating curve (AUC-ROC) of urine TIMP-2 multiplied by urine IGFBP7 ([TIMP-2]*[IGFBP7]) was 0.80, which is superior to that of other biomarkers, including NGAL, KIM-1, IL-18 as well as urine TIMP-2 and urine IGFBP7 independently. In several reports, the AUC-ROC of urine [TIMP-2]*[IGFBP7] for diagnosis of AKI ranged from 0.706 to 0.971 [12–21]. The aim of this meta-analysis was to synthesize these studies to evaluate the diagnostic value of urine [TIMP-2]*[IGFBP7] as an early biomarker of AKI.

## Methods

### Data sources and search strategy

Studies were identified by a literature search of the PubMed (http://www.ncbi.nlm.gov/pubmed), EMBASE (http://www.embase.com/) and Cochrane Library (http://www.cochranelibrary.com/) databases from inception to October 2, 2016, with the following keywords and Medical Subject Heading (MeSH) terms “acute kidney injury” or “AKI” and “TIMP-2” and “IGFBP7” (see S2 Text for details of the search strategies). The search strategies were based on MeSH terms combined with free text terms. In addition, the reference lists of the retrieved articles were reviewed to identify additional studies. The searches were performed independently by two investigators (YS and YW).

### Study selection

Clinical studies in English of urine [TIMP-2]*[IGFBP7] as biomarkers of AKI were chosen and a 2×2 quadrant table was created. Two reviewers (YS and YW) reviewed the titles and abstracts of all citations and then read the full text of articles deemed relevant.

**Fig 1:**
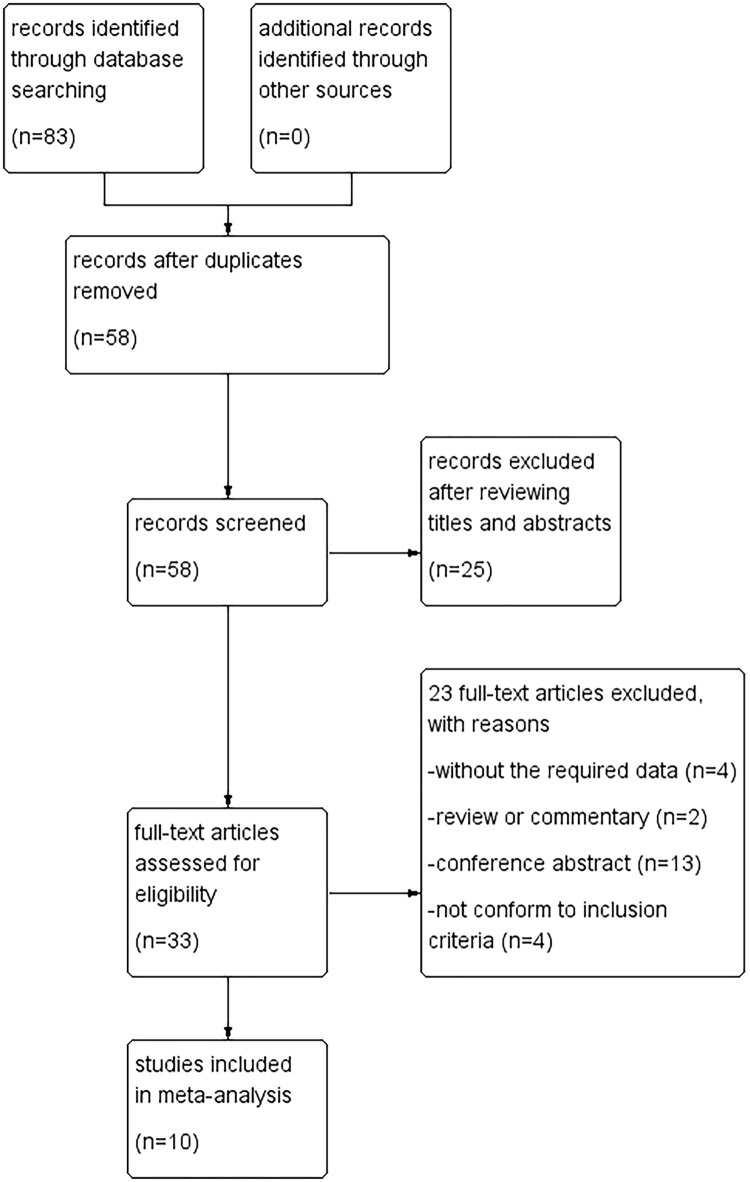
Flow chart of study selection.

According to inclusion and exclusion criteria, the primary search retrieved 83 studies from databases mentioned above. Of these, 25 articles were excluded because of duplication. After browsing the titles and abstracts, 25 additional articles were excluded. After reading each of the remaining publications in detail, 10 prospective cohort studies[12–21] were finally selected (Fig 1).

### Data extraction and quality assessment

The following information was extracted by two reviewers (YS and YW): first author, publication year, country of origin, study design, sample size, population setting, patient age, diagnostic criteria for AKI, blinded or not, method for detection of TIMP-2 and IGFBP7, time of detection, cut-off point, true-positive rate, false-positive rate, false-negative rate, and true-negative rate. When there were several cut-off points in one article, the time recommended in the conclusion or that which considered both summary sensitivity and specificity was chosen. If the time of detection was not clearly expressed, the latest time of specimen collection mentioned in the articles was used.

The methodological quality of each study was independently evaluated by two authors (YS and YT). This meta-analysis employed a quality assessment tool, with QUADAS-2 as a standard[22], which included 11 questions (scored as yes, no, or unclear), was specifically developed for systematic reviews of diagnostic accuracy studies to assess study bias.

### Data synthesis and analysis

Meta-Disc (ver. 1.4) software for meta-analysis of test accuracy data was employed in this study. Heterogeneity in meta-analysis indicates the degree of variability in results across studies and was appraised using the Q test, *p* value, and I^2^ index, which revealed thresholds for low (I^2^<50%), moderate (50%≤ I^2^<75%), and high (I^2^≥75%) heterogeneity. Sensitivity, specificity, positive and negative likelihood ratios, diagnostic odds ratios, and SROC curves were summarized using a random effects model with the DerSimonian and Liard procedure. A useful predictor of AKI risk was defined as an AUC-ROC of>0.7. The Spearman correlation coefficient of logarithmic sensitivity and logarithm (1-specificity) was used to test the presence of a threshold effect.

## Results

### Search results and study characteristics

10 prospective cohort studies [12–21] were selected, which were all published from 2014 to 2016 and included a total of 1709 patients, 328 of whom developed AKI. The characteristics of each of the studies are listed in Table 1. The study cohorts included patients accepted for cardiac surgery and ICU patients. One study enrolled infants. As shown in Table 1, the definition of AKI varied among the studies.

**Table 1:** Characteristics of studies included in this meta-analysis.

study	country	Study design	Sample size/total	AKI	Non-AKI	Population setting	age	AKI definition	Blind method
Hoste[12], 2014	USA	Multi-center prospective cohort study	153/1709	61	92	ICU	AKI 64(54, 75) NAKI 65(54, 78)	Achieve KDIGO stage 2–3 in 12 hours	yes
Wetz[13], 2015	Germany	Prospective cohort study	42/1709	16	26	CABG	AKI 66.5(61, 73) NAKI 75(72, 81)	KDIGO	unknown
Meersch[14], 2014	Germany	Prospective cohort study	50/1709	26	24	CPB	AKI 70±12 NAKI 72±11	KDIGO	unknown
Meersch[15], 2014	Germany	Prospective cohort study	51/1709	12	39	CPB	AKI 1.5±1 NAKI 3±0.5	pRIFLE	yes
Bihorac[16], 2014	USA	Multi-center prospective cohort study	408/1709	71	337	ICU	AKI 62±16 NAKI 63±17	Achieve KDIGO stage 2–3 in 12 hours	yes
Pilarczy[17], 2015	Germany	Prospective cohort study	60/1709	6	54	CABG	AKI 76.2±3.9 NAKI 68.8±9.1	Achieve KDIGO2-3 within 48 hours	yes
Dusse[18], 2016	Germany	Prospective cohort study	40/1709	15	25	TAVI	AKI 81.4±4.2 NAKI 80.7±5.9	KDIGO 2–3 within 48 hours	yes
Kimmel[19], 2016	Germany	Prospective cohort study	298/1709	46	252	ED	AKI 65±16 NAKI 63±14	KDIGO 2–3 within 12 hours	yes
Gunnerson[20], 2016	Europe and north America	Multicenter prospective cohort study	375/1709	35	340	Surgical ICU patients	AKI 67(13) NAKI 64(14)	KDIGO 2–3 and clinical adjudication for AKI	yes
Honore[21], 2016	Europe and north America	Multicenter prospective cohort study	232/1709	40	192	sepsis	AKI 64(16) NAKI 62(17)	KDIGO 2–3 and clinical adjudication for AKI	yes

AKI, acute kidney injury; AKIN, acute kidney injury network; CABG, coronary artery bypass grafting; CPB, cardiopulmonary bypass; ED, emergency department; ICU, intensive care unit; KDIGO, kidney disease: improving global outcomes; pRIFLE, pediatric risk, injury, failure, loss, end-stage renal disease; TAVI, transcatheter aortic valve implantation.

### Quality assessment

**Fig 2:**
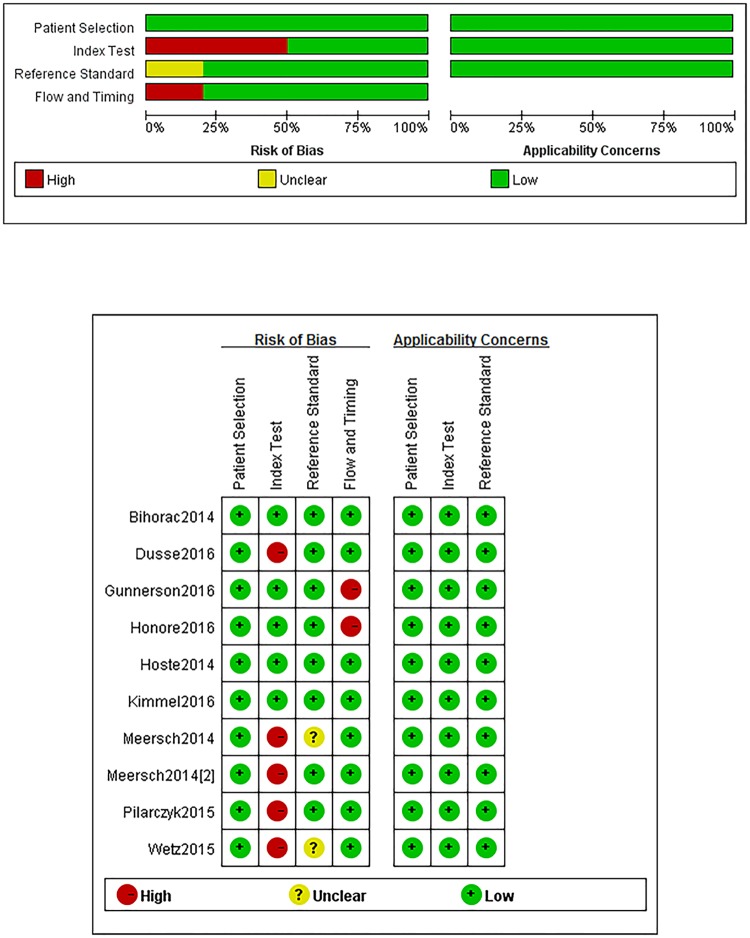
Methodological quality graph. Reviewer judgment of methodological quality of each individual study included in the analysis was performed using the Quality Assessment of Diagnostic Accuracy Studies (QUADAS-2) tool. “-” in red and “+” in green mean high risk and low risk respectively. “?” in yellow means unclear risk. In the index test part, some studies [13–15, 17–18] were valued as high risk, because these studies had not used setting threshold. Two studies [13–14] did not mentioned blind or not so we valued them unclear risk in the reference standard part. Two studies [20–21] was evaluated high risk in flow and timing part because of non-uniform diagnostic criteria used.

The Spearman correlation coefficient of these 10 articles was 0.116 (*p* = 0.751), suggesting no significant threshold effect. The quality of the studies according to QUADAS-2 tool is summarized in Fig 2. The quality of studies included was good. As there were only 10 studies included in the analysis, publication bias was not evaluated.

### Data synthesis

**Fig 3:**
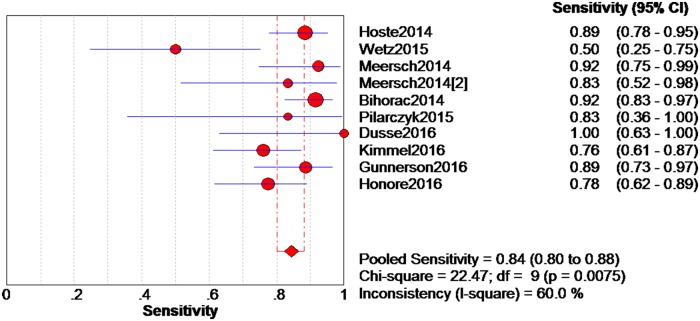
Forest plot of pooled sensitivity of urine [TIMP-2]*[IGFBP7]. The red spot and the horizontal lines represent the point estimate and the corresponding 95% confidence interval (CI), respectively. The red diamond and the red vertical lines represent the pooled estimate and the corresponding 95% CI. The different circle size means different weight of study.

**Fig 4:**
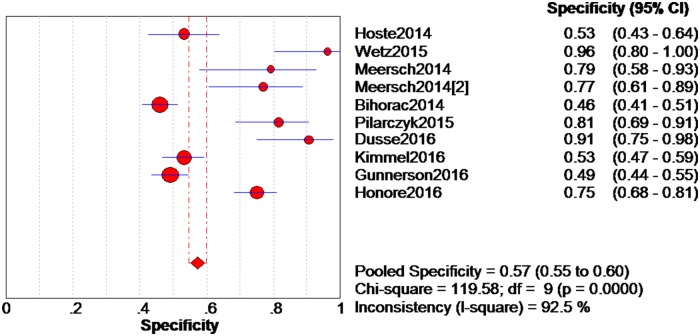
Forest plot of specificity of urine [TIMP-2]*[IGFBP7]. The red spot and the horizontal lines represent the point estimate and the corresponding 95% CI, respectively. The red diamond and the red vertical lines represent the pooled estimate and the corresponding 95% CI. The different circle size means different weight of study.

**Fig 5:**
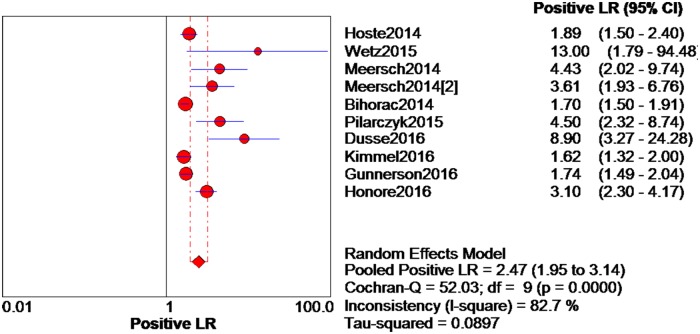
Forest plot of positive likelihood ratio of urine [TIMP-2]*[IGFBP7]. The red spot and the horizontal lines represent the point estimate and the corresponding 95% CI, respectively. The red diamond and the red vertical lines represent the pooled estimate and the corresponding 95% CI. The different circle size means different weight of study.

**Fig 6:**
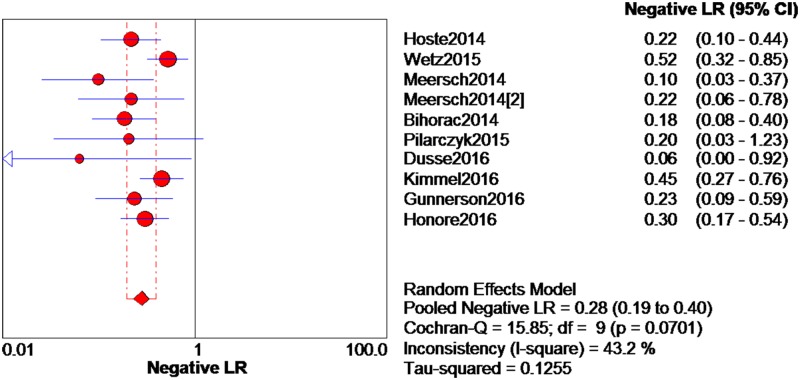
Forest plot of negative likelihood ratio of urine [TIMP-2]*[IGFBP7]. The red spot and the horizontal lines represent the point estimate and corresponding 95% CI, respectively. The red diamond and the red vertical lines represent the pooled estimate and the corresponding 95% CI. The different circle size means different weight of study. The arrow means the 95% confidence interval of study broader than the space provided.

**Fig 7:**
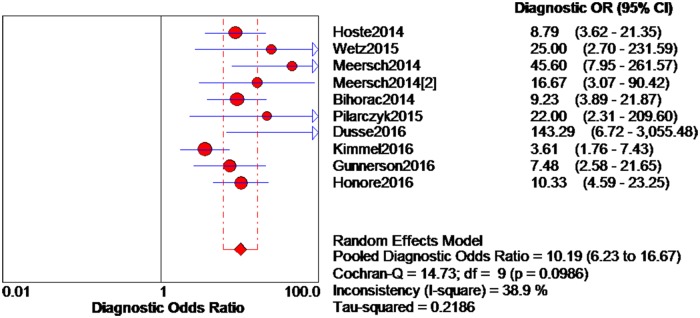
Forest plot of diagnostic odds ratio of urine [TIMP-2]*[IGFBP7]. The red spot and the horizontal lines represent the point estimate and the corresponding 95% CI, respectively. The red diamond and the red vertical lines represent the pooled estimate and the corresponding 95% CI. The different circle size means different weight of study. The arrow means the 95% confidence interval of study broader than the space provided.

**Fig 8:**
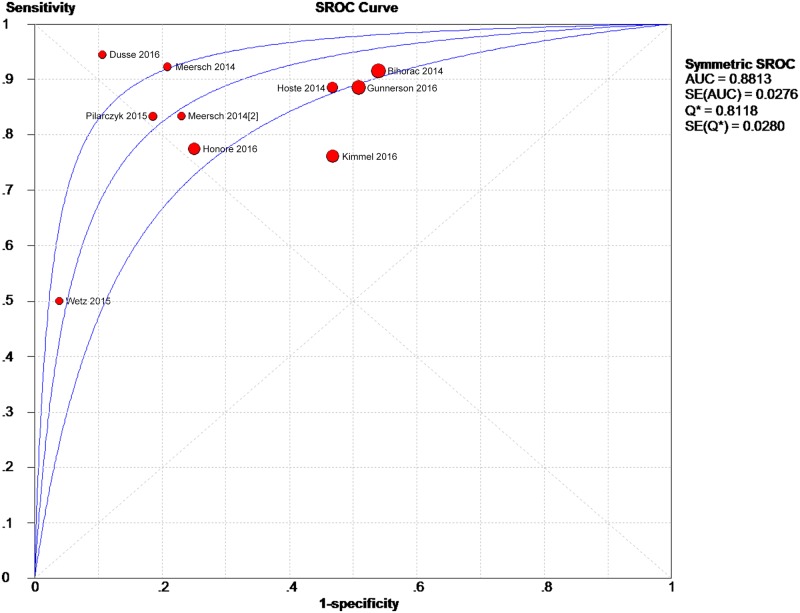
Summary receiver operating characteristic plot of [TIMP-2]*[IGFBP7] in predicting AKI. The red spots represent ten studies, the middle curve is a summary ROC curve, and the curves on the two sides represent the corresponding 95% confidence intervals. The different circle size means different weight of study.

The data extracted from the 10 eligible studies are shown in Table 2. The extracted data included true-negative, false-positive, false-negative, and true-negative rates, various cutoff values for urinary [TIMP-2]*[IGFBP7], sensitivities, specificities, AUC-ROC values, and time and method of TIMP-2 and IGFBP7 measurement. The estimated sensitivity of urinary [TIMP-2]*[IGFBP7] for the diagnosis of AKI was 0.84 (95% confidence interval (CI) = 0.80–0.88), the specificity was 0.57 (95% CI = 0.55–0.60), the positive likelihood ratio (PLR) was 2.47 (95% CI = 1.95–3.14), and the negative likelihood ratio (NLR) was 0.28 (95% CI = 0.19–0.40), with a diagnostic odds ratio (DOR) of 10.19 (95% CI = 6.23–16.67). I^2^ indexes were 60% for sensitivity, 92.5% for specificity, 82.7% for PLR, 43.2% for NLR, and 38.9% for DOR. Forest plots of sensitivity, specificity, positive likelihood ratio, negative likelihood ratio and diagnostic odds ratio are shown in Figs 3–7. SROC results showed that the AUC of urinary [TIMP-2]*[IGFBP7] was 0.8813, suggesting considerable efficiency of [TIMP-2]*[IGFBP7] for AKI diagnosis (Fig 8).

**Table 2:** Performance of urine [TIMP-2]*[IGFBP7] in the meta-analysis.

study	Time of measurement	Assay method	Cut-off point	No. of patients				sen	spe	AUC-ROC
				TP	FP	FN	TN			
Hoste[12],2014	Instantly when enrolled	NephroCheck Test	0.3	54	43	7	49	89%	53%	0.79
Wetz[13],2015	8 am on the first postoperative day	NephroCheck Test	1.065	8	1	8	25	47%	96%	0.706
Meersch[14],2014	First 24 hours following surgery	NephroCheck Test	0.5	24	5	2	19	92%	81%	0.84
Meersch[15],2014	4 hours after surgery	NephroCheck Test	0.7	10	9	2	30	83%	77%	0.85
Bihorac[16],2014	Instantly when enrolled	NephroCheck Test	0.3	65	182	6	155	92%	46%	0.82
Pilarczyk[17],2015	1 day after surgery	NephroCheck Test	0.89	5	10	1	44	80%	81%	0.869
Dusse[18],2016	Day 1 after TAVI	NephroCheck Test	1.03	8	3	0	29	100%	90%	0.971
Kimmel[19],2016	Within 27 hours after enrollment	NephroCheck Test	0.3	35	118	11	134	76%	53%	0.76
Gunnerson[20],2016	Within 12 hours	NephroCheck Test	0.3	31	173	4	167	89%	49%	0.84
Honore[21],2016	Within 12 hours	NephroCheck Test	1.0	31	48	9	144	77.5%	75%	0.84

FN, false negative; FP, false positive; sen, sensitivity; spe, specificity; TN, true negative; TP, true positive.

### Subgroup analysis

To evaluate the performance of urine [TIMP-2]*[IGFBP7] for AKI diagnosis in different population, we did subgroup analysis.

As shown in Table 1, nine studies included adults and one included infants. Since one of the ten studies enrolled infants, only an adult subgroup was used for analysis. The sensitivity and specificity of the adult subgroup were almost the same as the summary outcomes. The AUC-ROC of the adult subgroup was 0.8477.

The main populations of the included studies were patients who underwent cardiac surgery and those with serious internal medicine diseases. The subgroup analysis results are presented in Table 3. As indicated, the outcome of the cardiac surgery subgroup was much better than that of the ICU subgroup with DOR of 17.76 and AUC of 0.8897.

**Table 3:** Subgroup analysis of [TIMP-2]*[IGFBP7] in predicting AKI.

studies		sensitivity(95%CI)	specificity(95%CI)	PLR(95%CI)	NLR(95%CI)	DOR(95%CI)	AUC
**all**		0.84(0.80–0.88)	0.57(0.55–0.60)	2.47(1.95–3.14)	0.28(0.19–0.40)	10.19(6.23–16.67)	0.8813
**I**^**2**^**(%)**		60%	92.5%	82.7%	43.2%	38.9%	
**age**	adult(9)	0.84(0.80–0.88)	0.57(0.54–0.59)	2.37(1.86–3.02)	0.28(0.19–0.41)	9.98(5.90–16.87)	0.8477
	I^2^(%)	64.4%	92.9%	82.9%	48.5%	43.4%	
**population setting**	cardiac surgery(6)	0.83(0.75–0.90)	0.61(0.57–0.65)	4.31(1.98–9.38)	0.23(0.11–0.49)	17.76(8.31–37.92)	0.8897
	I^2^(%)	66.4%	92.9%	88.7%	60.2%	10.4%	
	ICU(4)	0.85(0.79–0.89)	0.55(0.52–0.59)	1.96(1.55–2.47)	0.29(0.19–0.43)	7.15(4.28–11.97)	0.7829
	I^2^(%)	59.9%	93.2%	80.7%	36.9%	31.7%	
**criterion**	all stage(3)	0.78(0.64–0.88)	0.83(0.74–0.99)	4.19(2.60–6.74)	0.25(0.07–0.83)	26.62(9.16–77.33)	0.9088
	I^2^(%)	80.2%	64%	0%	76.3%	0%	
	stage2-3(7)	0.86(0.81–0.90)	0.56(0.53–0.58)	2.17(1.74–2.71)	0.28(0.20–0.38)	8.17(4.98–13.41)	0.8190
	I^2^(%)	42%	93%	82.3%	8.9%	35.8%	

AUC, area under the receiver operating characteristic curve; CI, confidence interval; DOR, diagnostic odds ratio; ICU, intensive care unit; NLR, negative likelihood ratio; PLR, positive likelihood ratio.

The 10 studies adopted different diagnostic criteria. Three studies [13–15] used all stages of KDIGO/pRIFLE as AKI diagnosis criteria and other studies adopted stage 2–3 AKIN or KDIGO criteria to define AKI. As different AKI definitions might induce discrepancy, these studies were divided into two subgroups (all stages and stage 2–3 AKI), as shown in Table 3. The results demonstrated that urine [TIMP-2]*[IGFBP7] performed better in the subgroup of all stages than in the stage 2–3 subgroup with DOR of 26.62, AUC-ROC of 0.9088.

## Discussion

Early treatment can improve the prognosis or even reverse the acute renal damage in AKI. Various biomarkers of AKI have been evaluated in recent years, including NGAL, KIM-1, L-FABP, and CysC, among others. These recently identified biomarkers seem to behave better as diagnostic values during the early stage of AKI than more conventional biomarkers in some studies, but there are some disadvantages. For example, a study by Kooiman et al. [23] found that KIM-1 and NGAL did not fluctuate in 511 patients monitored by post-intravenous contrast-enhanced computed tomography, including those with contrast-induced AKI (CI-AKI). This result raises concern about the accuracy of NGAI and KIM-1 in CI-AKI. In addition, NGAL is reportedly influenced by many other common clinical factors, including baseline renal function, inflammation, pregnancy, cancer, etc. [24–27]. IL-18 was reported to be affected by inflammation [28]. Furthermore, the concentrations of NGAL, KIM-1, IL-18 and L-FABP are higher in patients with chronic kidney disease than healthy controls [29–35], which might interfere in the differentiation between AKI and chronic kidney disease. Hence, these biomarkers are not sufficient to differentially diagnose AKI.

TIMP-2 and IGFBP7 both play important roles in blocking stage G1 of the renal tubular epithelial cell cycle in the early stage of AKI. With involvement in the early pathophysiological changes of AKI, TIMP-2 and IGFBP7 are considered as promising candidate early biomarkers of AKI. Kashani et al. [11] evaluated the utility of more than 300 urine biomarkers of AKI performance and found that the most accurate was urine [TIMP-1]*[IGFBP7]. At present, several other clinical trials regarding urine [TIMP-2]*[IGFBP7] as a diagnostic indicator of AKI by different etiologies are underway. In the present study, a meta-analysis was conducted to further evaluate the diagnostic values of urine TIMP-2 times IGFBP7 in AKI. Part of the work of this meta-analysis has been reported as an abstract [36].

The synthesized sensitivity and specificity of the 10 identified studies in this meta-analysis were 0.84 and 0.57, respectively. The AUC-ROC was 0.8813, indicating that urine [TIMP-2]*[IGFBP7] had a reliable diagnostic accuracy for AKI.

To further investigate the value of [TIMP-2]*[IGFBP7] for diagnosis of AKI, among different patient populations and different ages, several subgroup analyses were performed. As shown in Table 3, ICU patients have diverse cause of AKI and more complications, which increases the risk of exposure to potential confounding factors, which may explain why urine [TIMP-2]*[IGFBP7] was more accurate in cardiac surgery patients, who usually have a relatively simple pathogenesis.

The AKIN, RIFLE and KDIGO criteria are dependent on serum creatinine and urine output, although neither is considered a gold standard. So, at present, there is no uniform diagnostic criterion for use in clinic trials. In this meta-analysis, each of the 10 included studies used different criteria. The results of this meta-analysis showed that the KDIGO/pRIFLE criteria had an AUC-ROC value of 0.9088 for all stages of AKI with good sensitivity. The specificity was much better than any other subgroup, suggesting that urine [TIMP-2]*[IGFBP7] performed well even in the early stage of AKI. However, this subgroup only contained three studies with relatively small sample sizes, thus this result might not be very reliable.

The results of adult subgroup analyses according to patient age showed a very similar AUC to that of the overall analysis. Considering only one study recruited non-adults, this subgroup design might not be appropriate. The time point of detection ranged from the time of enrollment to about 1 day which suggests that urine [TIMP-2]*[IGFBP7] was able to predict AKI in the very early period after injury onset.

The summary specificity of this meta-analysis was 0.57, which is not very good. So, [TIMP-2]*[IGFBP7] may be more useful to rule-out AKI.

There were some limitations to this study that should be addressed. First, only articles published in English were included, thus language bias inevitable. In the process of searching the databases, several studies without a 2×2 quadrant table were excluded. Among these, one study [37] had less optimistic results (small sample size, patients number≤15, and lack of definite information), thus some studies with unpublished negative results were omitted. As shown in Table 1, all of the included studies were conducted in Europe, especially in Germany, or North America. Besides, the study populations were restricted to patients selected for cardiac surgery and those with severe diseases. So, further studies with other etiologies, such as CI-AKI, other races, and larger cohorts are needed to verify the value of [TIMP-2]*[IGFBP7] for the diagnosis of AKI. Also, oliguria might impede urine sampling, which prevented the assessment of urine TIMP-2 and IGFBP7. Subsequent studies should also consider other possible interfering factors of urine TIMP-2 and IGFBP7.

## Conclusion

The result of this meta-analysis demonstrated that the AUC-ROC for diagnostic accuracy of urine [TIMP-2]*[IGFBP7] for AKI was good. The summary AUC-ROC, sensitivity and specificity of this meta-analysis were 0.8813, 0.84 and 0.57, respectively.

## Supporting Information

S1 Text PRISMA statement of the meta-analysis. (DOC)

S2 Text Search strategy. (DOCX)

S1 Table Weight of the studies. (DOCX)

## Abbreviations

AKI: acute kidney injury
AKIN: acute kidney injury network
AUC-ROC: area under the receiver operating characteristic curve
CABG: coronary artery bypass grafting
CI: confidence interval
CI-AKI: contrast-induced acute kidney injury
CPB: cardiopulmonary bypass
CysC: cystatin C
DOR: diagnostic odds ratio
ED: emergency department
FN: false negative
FP: false positive
ICU: intensive care unit
IGFBP7: insulin-like growth factor binding protein 7
IL-18: interleukin-18
KDIGO: kidney disease improving global outcomes
KIM-1: kidney injury molecule-1
MeSH: Medical Subject Heading
NGAL: neutrophil gelatinase-associated lipocalin
NLR: negative likelihood ratio
PLR: positive likelihood ratio
pRIFLE: pediatric risk, injury, failure, loss, end-stage renal disease
QUADAS-2: quality assessment for studies of diagnostic accuracy studies 2
sen: sensitivity
spe: specificity
TAVI: transcatheter aortic valve implantation
TIMP-2: issue inhibitor of metalloproteinase 2
TN: true negative
TP: true positive

## References

[1] Esson ML, Schrier RW. Diagnosis and treatment of acute tubular necrosis. Annals of internal medicine. 2002;137(9):744–52. 12416948 10.7326/0003-4819-137-9-200211050-00010

[2] Uchino S, Kellum JA, Bellomo R, Doig GS, Morimatsu H, Morgera S, et al Acute renal failure in critically ill patients: a multinational, multicenter study. Jama. 2005;294(7):813–8. 10.1001/jama.294.7.813 16106006

[3] Schrier RW, Wang W, Poole B, Mitra A. Acute renal failure: definitions, diagnosis, pathogenesis, and therapy. The Journal of clinical investigation. 2004;114(1):5–14. 10.1172/JCI22353 15232604 PMC437979

[4] Li PK, Burdmann EA, Mehta RL, World Kidney Day Steering C. Acute kidney injury: global health alert. Transplantation. 2013;95(5):653–7. 10.1097/TP.0b013e31828848bc 23503499

[5] Bagshaw SM, George C, Dinu I, Bellomo R. A multi-centre evaluation of the RIFLE criteria for early acute kidney injury in critically ill patients. Nephrology, dialysis, transplantation: official publication of the European Dialysis and Transplant Association—European Renal Association. 2008;23(4):1203–10. 10.1093/ndt/gfm744 17962378

[6] Coca SG, Yalavarthy R, Concato J, Parikh CR. Biomarkers for the diagnosis and risk stratification of acute kidney injury: a systematic review. Kidney international. 2008;73(9):1008–16. 10.1038/sj.ki.5002729 18094679

[7] Bellomo R, Kellum JA, Ronco C. Acute kidney injury. Lancet. 2012;380(9843):756–66. 10.1016/S0140-6736(11)61454-2 22617274

[8] Price PM, Safirstein RL, Megyesi J. The cell cycle and acute kidney injury. Kidney international. 2009;76(6):604–13. 10.1038/ki.2009.224 19536080 PMC2782725

[9] Boonstra J, Post JA. Molecular events associated with reactive oxygen species and cell cycle progression in mammalian cells. Gene. 2004;337:1–13. 10.1016/j.gene.2004.04.032 15276197

[10] Rodier F, Campisi J, Bhaumik D. Two faces of p53: aging and tumor suppression. Nucleic acids research. 2007;35(22):7475–84. 10.1093/nar/gkm744 17942417 PMC2190721

[11] Kashani K, Al-Khafaji A, Ardiles T, Artigas A, Bagshaw SM, Bell M, et al Discovery and validation of cell cycle arrest biomarkers in human acute kidney injury. Critical care (London, England). 2013;17(1):R25. Epub 2013/02/08. 10.1186/cc12503 PMC4057242 23388612

[12] Hoste Eric A., M PA, Kashani Kianoush, Chawla Lakhmir S., Joannidis Michael, Shaw Andrew D., Feldkamp Thorsten, Uettwiller-Geiger Denise L., McCarthy Paul, Shi Jing, Walker Michael G., Kellum John A. on behalf of the Sapphire Investigators. Derivation and validation of cutoffs for clinical use of cell cycle arrest biomarkers. Nephrology, dialysis, transplantation: official publication of the European Dialysis and Transplant Association—European Renal Association. 2014;29:2054–61. 10.1093/ndt/gfu292 PMC4209880 25237065

[13] Wetz AJ, Richardt EM, Wand S, Kunze N, Schotola H, Quintel M, et al Quantification of urinary TIMP-2 and IGFBP-7: an adequate diagnostic test to predict acute kidney injury after cardiac surgery? Critical care (London, England). 2015;19:3. Epub 2015/01/07. 10.1186/s13054-014-0717-4 PMC4310039 25560277

[14] Meersch Melanie S C, Van Aken Hugo, Martens Sven, Rossaint Jan, Singbartl Kai, Gorlich Dennis, Kellum John A., Zarbock Alexander. Urinary TIMP-2 and IGFBP7 as Early Biomarkers of Acute Kidney Injury and Renal Recovery following Cardiac Surgery. PloS one. 2014;9(3):e93460 10.1371/journal.pone.0093460 24675717 PMC3968141

[15] Meersch M, Schmidt C, Van Aken H, Rossaint J, Gorlich D, Stege D, et al Validation of cell-cycle arrest biomarkers for acute kidney injury after pediatric cardiac surgery. PloS one. 2014;9(10):e110865 Epub 2014/10/25. 10.1371/journal.pone.0110865 25343505 PMC4208780

[16] Bihorac A, Chawla LS, Shaw AD, Al-Khafaji A, Davison DL, Demuth GE, et al Validation of cell-cycle arrest biomarkers for acute kidney injury using clinical adjudication. American journal of respiratory and critical care medicine. 2014;189(8):932–9. 10.1164/rccm.201401-0077OC 24559465

[17] Pilarczyk K, Edayadiyil-Dudasova M, Wendt D, Demircioglu E, Benedik J, Dohle DS, et al Urinary [TIMP-2]*[IGFBP7] for early prediction of acute kidney injury after coronary artery bypass surgery. Annals of intensive care. 2015;5(1):50 Epub 2015/12/17. 10.1186/s13613-015-0076-6 26669781 PMC4679715

[18] Dusse F, Edayadiyil-Dudasova M, Thielmann M, Wendt D, Kahlert P, Demircioglu E, et al Early prediction of acute kidney injury after transapical and transaortic aortic valve implantation with urinary G1 cell cycle arrest biomarkers. BMC Anesthesiol. 2016;16:76 Epub 2016/09/10. 10.1186/s12871-016-0244-8 27609347 PMC5016985

[19] Kimmel M, Shi J, Latus J, Wasser C, Kitterer D, Braun N, et al Association of Renal Stress/Damage and Filtration Biomarkers with Subsequent AKI during Hospitalization among Patients Presenting to the Emergency Department. Clinical journal of the American Society of Nephrology: CJASN. 2016;11(6):938–46. Epub 2016/03/31. 10.2215/CJN.10551015 27026519 PMC4891754

[20] Gunnerson KJ, Shaw AD, Chawla LS, Bihorac A, Al-Khafaji A, Kashani K, et al TIMP2•IGFBP7 biomarker panel accurately predicts acute kidney injury in high-risk surgical patients. Journal of Trauma and Acute Care Surgery. 2016;80(2):243–9. 10.1097/TA.0000000000000912 26816218 PMC4729326

[21] Honore PM, Nguyen HB, Gong M, Chawla LS, Bagshaw SM, Artigas A, et al Urinary Tissue Inhibitor of Metalloproteinase-2 and Insulin-Like Growth Factor-Binding Protein 7 for Risk Stratification of Acute Kidney Injury in Patients with Sepsis. Critical Care Medicine. 2016;44(10):1851–60. 10.1097/CCM.0000000000001827 27355527 PMC5089124

[22] Whiting PF, Rutjes AW, Westwood ME, Mallett S, Deeks JJ, Reitsma JB, et al QUADAS-2: a revised tool for the quality assessment of diagnostic accuracy studies. Annals of internal medicine. 2011;155(8):529–36. 10.7326/0003-4819-155-8-201110180-00009 22007046

[23] Kooiman J, van de Peppel WR, Sijpkens YW, Brulez HF, de Vries PM, Nicolaie MA, et al No increase in Kidney Injury Molecule-1 and Neutrophil Gelatinase-Associated Lipocalin excretion following intravenous contrast enhanced-CT. European radiology. 2015;25(7):1926–34. 10.1007/s00330-015-3624-4 25773936 PMC4457910

[24] McIlroy DR, Wagener G, Lee HT. Neutrophil gelatinase-associated lipocalin and acute kidney injury after cardiac surgery: the effect of baseline renal function on diagnostic performance. Clinical journal of the American Society of Nephrology: CJASN. 2010;5(2):211–9. 10.2215/CJN.04240609 20056755 PMC2827603

[25] Lippi G, Meschi T, Nouvenne A, Mattiuzzi C, Borghi L. Neutrophil gelatinase-associated lipocalin in cancer. Advances in clinical chemistry. 2014;64:179–219. 24938019 10.1016/b978-0-12-800263-6.00004-5

[26] Lindberg S, Jensen JS, Mogelvang R, Pedersen SH, Galatius S, Flyvbjerg A, et al Plasma neutrophil gelatinase-associated lipocalinin in the general population: association with inflammation and prognosis. Arteriosclerosis, thrombosis, and vascular biology. 2014;34(9):2135–42. 10.1161/ATVBAHA.114.303950 24969771

[27] Odum L, Andersen AS, Hviid TV. Urinary neutrophil gelatinase-associated lipocalin (NGAL) excretion increases in normal pregnancy but not in preeclampsia. Clin Chem Lab Med. 2014;52(2):221–5. 10.1515/cclm-2013-0547 24108204

[28] Li Y, Li X, Zhou X, Yan J, Zhu X, Pan J, et al Impact of sepsis on the urinary level of interleukin-18 and cystatin C in critically ill neonates. Pediatric nephrology. 2013;28(1):135–44. 10.1007/s00467-012-2285-7 22918444

[29] Peters HP, Waanders F, Meijer E, van den Brand J, Steenbergen EJ, van Goor H, et al High urinary excretion of kidney injury molecule-1 is an independent predictor of end-stage renal disease in patients with IgA nephropathy. Nephrology, dialysis, transplantation: official publication of the European Dialysis and Transplant Association—European Renal Association. 2011;26(11):3581–8. 10.1093/ndt/gfr135 21467131

[30] Sabbisetti VS, Waikar SS, Antoine DJ, Smiles A, Wang C, Ravisankar A, et al Blood kidney injury molecule-1 is a biomarker of acute and chronic kidney injury and predicts progression to ESRD in type I diabetes. Journal of the American Society of Nephrology: JASN. 2014;25(10):2177–86. 10.1681/ASN.2013070758 24904085 PMC4178434

[31] Parikh CR, Dahl NK, Chapman AB, Bost JE, Edelstein CL, Comer DM, et al Evaluation of urine biomarkers of kidney injury in polycystic kidney disease. Kidney international. 2012;81(8):784–90. 10.1038/ki.2011.465 22258321 PMC3319327

[32] van Timmeren MM, van den Heuvel MC, Bailly V, Bakker SJ, van Goor H, Stegeman CA. Tubular kidney injury molecule-1 (KIM-1) in human renal disease. The Journal of pathology. 2007;212(2):209–17. 10.1002/path.2175 17471468

[33] Yong K, Ooi EM, Dogra G, Mannion M, Boudville N, Chan D, et al Elevated interleukin-12 and interleukin-18 in chronic kidney disease are not associated with arterial stiffness. Cytokine. 2013;64(1):39–42. 10.1016/j.cyto.2013.05.023 23778029

[34] Liang D, Liu HF, Yao CW, Liu HY, Huang-Fu CM, Chen XW, et al Effects of interleukin 18 on injury and activation of human proximal tubular epithelial cells. Nephrology. 2007;12(1):53–61. 10.1111/j.1440-1797.2006.00737.x 17295661

[35] Araki S, Haneda M, Koya D, Sugaya T, Isshiki K, Kume S, et al Predictive effects of urinary liver-type fatty acid-binding protein for deteriorating renal function and incidence of cardiovascular disease in type 2 diabetic patients without advanced nephropathy. Diabetes care. 2013;36(5):1248–53. 10.2337/dc12-1298 23223350 PMC3631864

[36] Su Y, Xie S, Zhong L, Wu Y, Tian Y, Liao X. Diagnostic value of urine [timp-2]$[IGFBP7] in AKI: A meta-analysis. Hong Kong Journal of Nephrology. 2015;17(2):S95.

[37] Roux-Morlon B, Tafer N, Boyer P, Sgro C, Mauriat P, Ouattara A. Dosage feasibility of urine biomarkers [TIMP-2]/[IGFBP7] in paediatric acute kidney injury after cardiac surgery with cardiopulmonary bypass. Applied Cardiopulmonary Pathophysiology. 2014;18:24–5.

