# Supplementary material for: Diagnostic Value of Urine Tissue Inhibitor of Metalloproteinase-2 and Insulin-Like Growth Factor-Binding Protein 7 for Acute Kidney Injury: A Meta-Analysis
Source: PLoS One. 2017 Jan 20;12(1):e0170214. doi: 10.1371/journal.pone.0170214 (PMC5249150; doi:10.1371/journal.pone.0170214)
Supplement: S2 Text — (DOCX) [file pone.0170214.s002.docx]

PUBMED

1. "Acute Kidney Injury" [tiab] OR "Kidney Injuries, Acute" [tiab] OR "Kidney Injuriy, Acute" [tiab] OR "Acute Renal Injury" [tiab] OR "Acute Renal Injuries" [tiab] OR "Renal Injuries, Acut"e[tiab] OR "Renal Injury, Acute" [tiab] OR "Renal Insufficiency, Acute" [tiab] OR "Acute Renal Insufficiencies" [tiab] OR "Renal Insufficiencies,Acute" [tiab] OR "Acute Renal Insufficiency" [tiab] OR "Kidney Insufficiency,Acute" [tiab] OR "Acute Kidney Insufficiencies" [tiab] OR "Kidney Insufficiencies, Acute" [tiab] OR "Acute Kidney Insufficiency" [tiab] OR "Kidney Failure, Acute" [tiab] OR "Acute Kidney Failures" [tiab] OR "Kidney Failures, Acute" [tiab] OR "Acute Renal Failure" [tiab] OR "Acute Renal Failures" [tiab] OR "Renal Failures, Acute" [tiab] OR "Renal Failure, Acute" [tiab] OR "Acute Kidney Failure" [tiab] 47778
2. "acute kidney injury"[Mesh] 37627
3. 1 OR 2 60059
4. "Tissue Inhibitor of Metalloproteinase-2" [Mesh] 3115
5. "Metalloproteinase-2 Tissue Inhibitor"[tiab] OR "Tissue Inhibitor of Metalloproteinase 2" [tiab] OR TIMP-2[tiab] 3885
6. 4 OR 5 4747
7. As we found only "insulin-like growth factor binding protein-related protein 1" as a supplementary concept which is different from the marker we discussed, we gave up the "MeSH Terms" of IGFBP7 search.
8. "insulin like-growth factor binding protein-7" [tiab] OR IGFBP-7[tiab] OR "IGFBP7 protein, human" [tiab] OR "insulin-like growth factor binding protein 7, human" [tiab]OR IGFBP7 340
9. 3 AND 6 AND 8 36

EMBASE

1. ‘Acute kidney failure’/exp 59701
2. ‘Acute Kidney Injuries’: ab, ti or ‘Kidney Injuries, Acute’: ab, ti or ‘Kidney Injury, Acute’: ab, ti or ‘Acute Renal Injury’: ab, ti or ‘Acute Renal Injuries’: ab, ti or ‘Renal Injuries, Acute’: ab, ti or ‘Renal Injury, Acute’: ab, ti or ‘Renal Insufficiency, Acute’: ab, ti or ‘Acute Renal Insufficiencies’: ab, ti or ‘Renal Insufficiencies, Acute’: ab, ti or ‘Acute Renal Insufficiency’: ab, ti or ‘Kidney Insufficiency, Acute’: ab, ti or ‘Acute Kidney Insufficiencies’ : ab, ti or ‘Kidney Insufficiencies, Acute’: ab, ti or ‘Acute Kidney Insufficiency’: ab, ti or ‘Kidney Failure, Acute’: ab, ti or ‘Acute Kidney Failures’: ab, ti or ‘Kidney Failures, Acute’: ab, ti or ‘Acute Renal Failure’: ab, ti or ‘Acute Renal Failures’: ab, ti or ‘Renal Failures, Acute’: ab, ti or ‘Renal Failure, Acute’: ab, ti 30560
3. 1 or 2 67246
4. ‘Tissue Inhibitor of Metalloproteinase 2’/exp or ‘Metalloproteinase-2 Tissue Inhibitor’ : ab, ti or ‘Tissue Inhibitor of Metalloproteinase-2’ : ab, ti or TIMP-2: ab, ti 7042
5. ‘insulin like-growth factor binding protein-7’ : ab, ti or ‘igfbp 7’ : ab, ti or ‘IGFBP7 protein, human’ : ab, ti or ‘insulin-like growth factor binding protein 7, human’ : ab, ti or ‘igfbp7’: ab, ti 429
6. 3 and 4 and 5 51
7. 6 not ('letter'/it OR 'review'/it) 45

COCHRANE

1. MeSH descriptor: [Acute Kidney Injury] explode all trees 884
2. Kidney or renal :ti,ab,kw (Word variations have been searched) 41135
3. Failure or injury or insufficiency :ti,ab,kw (Word variations have been searched) 84127
4. Acute :ti,ab,kw (Word variations have been searched) 80036
5. #2 and #3 and #4 4014
6. #1 or #5 4034
7. MeSH descriptor: [Tissue Inhibitor of Metalloproteinase-2] explode all trees 39
8. Metalloproteinase-2 Tissue Inhibitor or Tissue Inhibitor of Metalloproteinase 2 or TIMP-2:ti,ab,kw (Word variations have been searched) : ti,ab,kw (Word variations have been searched) 232
9. #7 or #8 232
10. insulin like-growth factor binding protein-7 or IGFBP-7 or IGFBP7 protein, human or insulin-like growth factor binding protein 7, human: ti,ab,kw (Word variations have been searched) 310
11. #6 and #9 and #10 2
