# Supplementary material for: Diagnostic Value of Urine Tissue Inhibitor of Metalloproteinase-2 and Insulin-Like Growth Factor-Binding Protein 7 for Acute Kidney Injury: A Meta-Analysis
Source: PLoS One. 2017 Jan 20;12(1):e0170214. doi: 10.1371/journal.pone.0170214 (PMC5249150; doi:10.1371/journal.pone.0170214)
Supplement: S1 Table — (DOCX) [file pone.0170214.s003.docx]

**Table 1.** Weight of all studies

**Summary Diagnostic Odds Ratio (Random effects model)**

Study | DOR [95% Conf. Iterval.] % Weight

--------------------------------------------------------------------------------------------

Hoste2014 | 8.791 3.619 - 21.353 14.90

Wetz2015 | 25.000 2.699 - 231.59 4.18

Meersch2014 | 45.600 7.950 - 261.57 6.23

Meersch2014[2] | 16.667 3.072 - 90.423 6.56

Bihorac2014 | 9.226 3.891 - 21.874 15.30

Pilarczyk2015 | 22.000 2.309 - 209.60 4.10

Dusse2016 | 143.29 6.719 - 3055.5 2.38

Kimmel2016 | 3.613 1.757 - 7.432 17.83

Gunnerson2016 | 7.481 2.585 - 21.654 12.32

Honore2016 | 10.333 4.593 - 23.248 16.20

**Table 2**. Weight of studies in adult subgroup.

**Summary Diagnostic Odds Ratio (Random effects model)**

Study | DOR [95% Conf. Iterval.] % Weight

--------------------------------------------------------------------------------------------

Hoste2014 | 8.791 3.619 - 21.353 15.85

Wetz2015 | 25.000 2.699 - 231.59 4.67

Meersch2014 | 45.600 7.950 - 261.57 6.89

Bihorac2014 | 9.226 3.891 - 21.874 16.24

Pilarczyk2015 | 22.000 2.309 - 209.60 4.57

Dusse2016 | 143.29 6.719 - 3055.5 2.68

Kimmel2016 | 3.613 1.757 - 7.432 18.72

Gunnerson2016 | 7.481 2.585 - 21.654 13.25

Honore2016 | 10.333 4.593 - 23.248 17.13

--------------------------------------------------------------------------------------

**Table 3**. Weight of studies in surgery subgroup.

Summary Diagnostic Odds Ratio (Random effects model)

Study | DOR [95% Conf. Iterval.] % Weight

--------------------------------------------------------------------------------------------

Wetz2015 | 25.000 2.699 - 231.59 10.79

Meersch2014 | 45.600 7.950 - 261.57 16.79

Meersch2014[2] | 16.667 3.072 - 90.423 17.78

Pilarczyk2015 | 22.000 2.309 - 209.60 10.55

Dusse2016 | 143.29 6.719 - 3055.5 5.91

Gunnerson2016 | 7.481 2.585 - 21.654 38.18

**Table 4**.Weight of studies in ICU subgroup.

Summary Diagnostic Odds Ratio (Random effects model)

Study | DOR [95% Conf. Iterval.] % Weight

--------------------------------------------------------------------------------------------

Hoste2014 | 8.791 3.619 - 21.353 22.45

Bihorac2014 | 9.226 3.891 - 21.874 23.29

Kimmel2016 | 3.613 1.757 - 7.432 29.03

Honore2016 | 10.333 4.593 - 23.248 25.23

--------------------------------------------------------------------------------------------

**(REM) pooled DOR | 7.154 4.275 - 11.971**

**Table 5**. Weight of studies in all stage subgroup.

Summary Diagnostic Odds Ratio (Random effects model)

Study | DOR [95% Conf. Iterval.] % Weight

--------------------------------------------------------------------------------------------

Wetz2015 | 25.000 2.699 - 231.59 22.95

Meersch2014 | 45.600 7.950 - 261.57 37.28

Meersch2014[2] | 16.667 3.072 - 90.423 39.77

**Table 6**. Weight of studies in stage 2-3 subgroup.

**Summary Diagnostic Odds Ratio (Random effects model)**

Study | DOR [95% Conf. Iterval.] % Weight

--------------------------------------------------------------------------------------------

Hoste2014 | 8.791 3.619 - 21.353 17.99

Bihorac2014 | 9.226 3.891 - 21.874 18.57

Pilarczyk2015 | 22.000 2.309 - 209.60 4.33

Dusse2016 | 143.29 6.719 - 3055.5 2.46

Kimmel2016 | 3.613 1.757 - 7.432 22.39

Gunnerson2016 | 7.481 2.585 - 21.654 14.38

Honore2016 | 10.333 4.593 - 23.248 19.89
